# Structural and Functional Alterations in Right Dorsomedial Prefrontal and Left Insular Cortex Co-Localize in Adolescents with Aggressive Behaviour: An ALE Meta-Analysis

**DOI:** 10.1371/journal.pone.0136553

**Published:** 2015-09-04

**Authors:** Nora Maria Raschle, Willeke Martine Menks, Lynn Valérie Fehlbaum, Ebongo Tshomba, Christina Stadler

**Affiliations:** Department of Child and Adolescent Psychiatry, Psychiatric University Clinics Basel, Basel, Switzerland; Benito Menni Complejo Asistencial en Salud Mental, SPAIN

## Abstract

Recent neuroimaging work has suggested that aggressive behaviour (AB) is associated with structural and functional brain abnormalities in processes subserving emotion processing and regulation. However, most neuroimaging studies on AB to date only contain relatively small sample sizes. To objectively investigate the consistency of previous structural and functional research in adolescent AB, we performed a systematic literature review and two coordinate-based activation likelihood estimation meta-analyses on eight VBM and nine functional neuroimaging studies in a total of 783 participants (408 [224AB/184 controls] and 375 [215 AB/160 controls] for structural and functional analysis respectively). We found 19 structural and eight functional foci of significant alterations in adolescents with AB, mainly located within the emotion processing and regulation network (including orbitofrontal, dorsomedial prefrontal and limbic cortex). A subsequent conjunction analysis revealed that functional and structural alterations co-localize in right dorsomedial prefrontal cortex and left insula. Our results are in line with meta-analytic work as well as structural, functional and connectivity findings to date, all of which make a strong point for the involvement of a network of brain areas responsible for emotion processing and regulation, which is disrupted in AB. Increased knowledge about the behavioural and neuronal underpinnings of AB is crucial for the development of novel and implementation of existing treatment strategies. Longitudinal research studies will have to show whether the observed alterations are a result or primary cause of the phenotypic characteristics in AB.

## Introduction

Aggressive behaviour (AB), as observed in social disorders such as DBD (including conduct (CD) and oppositional defiant disorder (ODD)), is characterized by a repeated pattern of antisocial behaviour and severe aggression, where the basic rights of others, major age-appropriate norms or societal rules are violated [1]. Such problems can cause significant impairment in social, academic, or occupational functioning [2,3]. Clinical and subclinical forms of AB are observed in up to 14% of all girls and 16% of all boys [4]. The negative impact of aggression-related problems reaches beyond a patient’s family, ultimately affecting society as a whole (e.g. school-dropouts, delinquency, teen-pregnancies, substance abuse or difficulties integrating into work life [3,5,6]). Early conduct problems are key precursors of persistent AB and thus also predictive for ODD, CD and antisocial personality disorder in adulthood [7]. Neurodevelopmental theories [8,9,10] and longitudinal studies [11] are in line with these behavioural observations, suggesting that the presence of early brain alterations in individuals with aggressive behaviour may heighten the risk for long-lasting social impairments [12,13]. In the current paper we particularly focus on adolescents with *aggressive behaviour* (AB), hereby summarizing neuroimaging research in youths with either conduct problems, CD or ODD.

In recent years structural (e.g voxel-based/surface-based) and functional (e.g. fMRI/PET) neuroimaging techniques have grown into powerful tools to investigate the neuronal basis of the human brain in typically developing individuals as well as patients. It has been demonstrated that both, brain structure and function, may be modified by experience [14,15]. Activation-dependant structural plasticity can even occur after as little as seven days of training [16,17] and it is suggested to play a key role in human adaptation to environmental changes and disease. Even though neuroimaging evidence points toward a neuronal basis of AB [13,18], the overall number of research studies within this population remains relatively scarce. Furthermore, it has to be noted that AB characteristics as seen in CD and/or ODD are considered heterogeneous in respect to their pathologies. CD and ODD are frequently associated with comorbidities such as attention-deficit hyperactivity disorder (ADHD) or anxiety [19]). These comorbid disorders can differ in their pathophysiological mechanisms, some of them seem exclusive on a biological level making it possible that different developmental trajectories with varying neurobiological bases lead to the clinical manifestations of AB [20]. The vagueness of the group definition within many of the current studies on AB is thus bound to impact general conclusions drawn from it.

Even though the total number of studies is still limited, neuroanatomical and functional variations in youths with AB have been reported with increased frequency since the advent of modern neuroimaging. In particular, brain structure in AB has been investigated using voxel-based morphometry (VBM), diffusion tensor imaging (DTI) or surfaced-based morphometry. VBM studies for example have revealed differences in gray and white matter volume in brain regions including the amygdala, insula, orbitofrontal and dorsomedial prefrontal cortex (e.g. [21,22,23,24]) when comparing adolescents with AB and typically developing controls. Similarly, studies using surface-based morphometry [25,26] or DTI [27,28,29,30,31,32,33] provide evidence for structural alterations and/or impaired connectivity within brain regions involved in emotion processing, reward and empathy. Functional neuroimaging studies corroborate the structural neuroimaging literature. Cognitive paradigms employed in the investigation of AB have focused on disturbances in the emotion processing and regulation network of the brain. These tasks particularly target emotion processing/regulation [34,35,36,37,38,39,40,41,42,43], empathy [41,44,45], theory of mind [46], passive avoidance [47], decision making [48,49] or executive functioning [40,42,50]. Overall, studies point towards aberrant brain function in AB in key areas of social cognition and emotion, including prefrontal (orbitofrontal, dorsolateral and medial prefrontal cortex), limbic (e.g. amygdala, anterior insula, cingulate cortex) and temporal cortices.

Despite increasing evidence about the uniformity of atypical brain structure and function in AB, it has yet to be objectively determined which brain regions are commonly affected. Functional and structural neuroimaging studies are crucial for the understanding of the phenotype and aetiology of AB. However, most results and interpretations are based on individual neuroimaging studies and present various limitations (e.g. small sample sizes, low reliability, dependency on task chosen [51,52,53]). Furthermore, very few imaging studies have yet investigated brain structure and function in the same population. Activation likelihood estimation (ALE) meta-analyses allow the identification of consistent findings of brain activation and structure across multiple data sets. Hereby, ALE quantitatively investigates communalities between reported foci based on modelling them as probability distributions centered around the corresponding coordinates. The resulting probability maps mirror the likelihood of morphological change and/or activation on a voxel-wise level across an entire set of studies [51]. ALE has been successfully applied in meta-analyses of various neuropsychiatric disorders to date [54,55,56,57,58] and provides a promising tool for a more unified investigation of pathophysiologic changes in disease.

Therefore, the present paper intends to close this gap in research and aims to aggregate all structural and functional neuroimaging studies conducted in adolescent AB to date. In a first step, we planned to conduct a systematic literature review of neuroimaging findings in adolescents with AB. Secondly two separate meta-analyses looking at gray matter volume reductions as well as hypoactivations during emotion processing tasks in AB were carried out. Finally, we decided to run a conjunction analysis to identify potential overlaps in deviant brain structure and function in adolescents with AB.

## Method

### Participants

We decided to focus our analysis on adolescents with *aggressive behaviour* (AB) in general as opposed to a specific clinical diagnosis. By including both community samples and clinical samples in the present meta-analyses we adhere to the heterogeneity in juvenile aggression. This heterogeneity is further reflected by different behavioural symptoms of aggression and antisocial tendencies, such as oppositional behaviour, impulsive hot-tempered quarrels or premeditated violent acts, the presence of callous unemotional/psychopathic traits or co-morbid conditions in CD and ODD patients. All studies were conducted during childhood and/or adolescence and share the communality of aggression and antisocial tendencies within the populations studied. Thus, AB as defined here may be considered an umbrella term for children and adolescents with a range of subclinical and clinically relevant symptoms of pathological aggression.

### Study Selection

**Fig 1:**
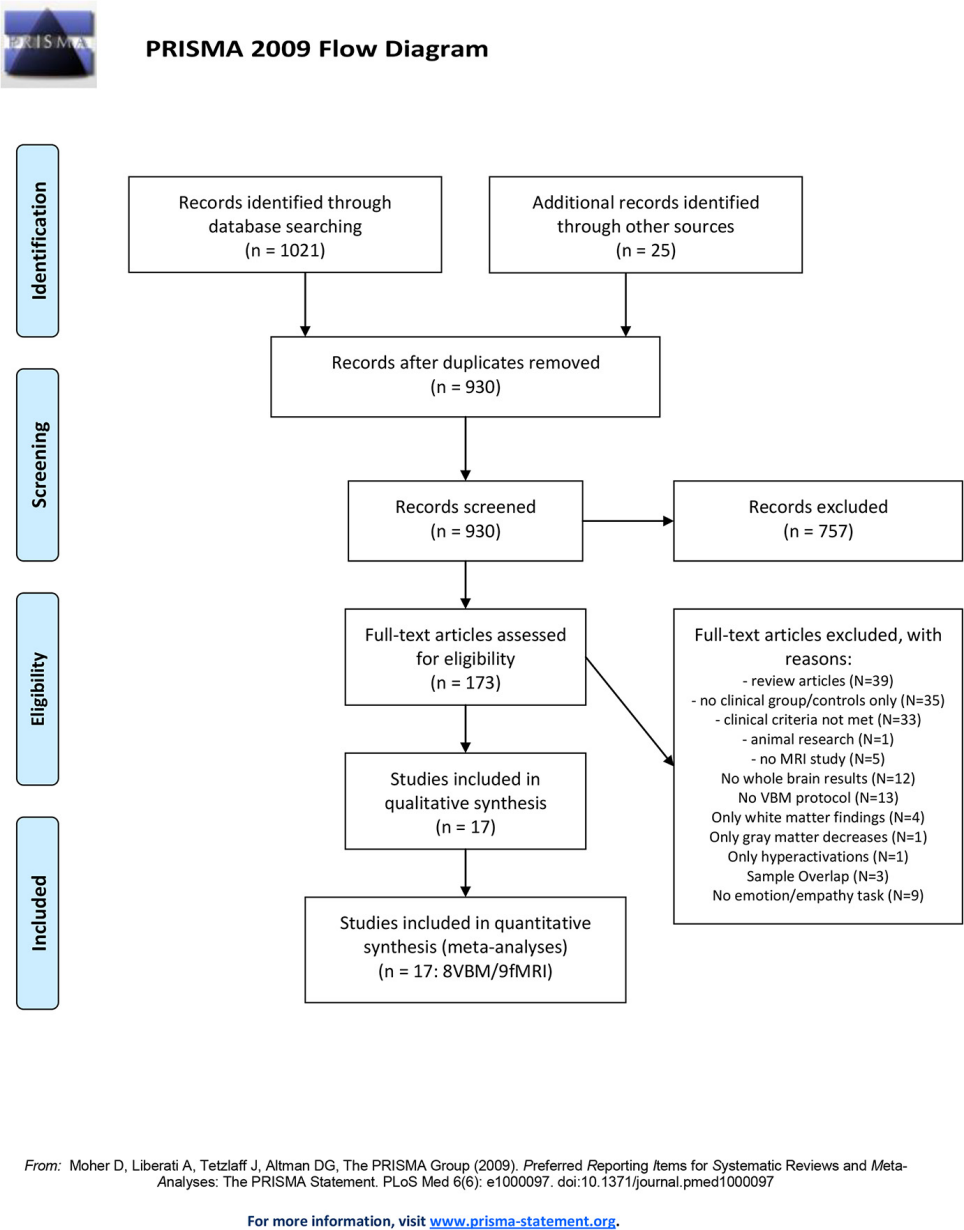
Systematic literature research. Literature research according to the Preferred Reporting Items for Systematic Reviews and Meta-Analyses (PRISMA) guidelines and the revised Quality Of Reporting Of Meta-analyses (QUOROM) statement (59) resulting in 17 neuroimaging studies included in the current meta-analyses.

For the structural and functional neuroimaging meta-analyses we used PubMed and Google Scholar to systematically search for neuroimaging literature in AB. Literature searches were conducted and reviewed by several research team members (NMR, WMM, LVF, ET) and adhered to the Preferred Reporting Items for Systematic Reviews and Meta-Analyses (PRISMA; S1 Table) guidelines and the revised Quality Of Reporting Of Meta-analyses (QUOROM) statement [59]. Our main search (see Fig 1) conducted through PubMed included the following key words: *“conduct disorder”*, *“conduct problems”*, *“disruptive behaviour disorder”*, *“oppositional defiant disorder”* and *“aggression”*, each in combination with methodologically relevant terms including *“VBM”*, *“fMRI”* and/or *“neuroimaging”*. Moreover, a number of review articles published on conduct disorder, antisocial behaviour and aggression in adolescents were considered (e.g. [11,60,61,62,63,64,65]). Finally, additional publications were explored by searching the reference list of the articles obtained to assure integration of all data available. Studies were included in our meta-analyses if the following criteria were given: (**I**) included at least one clinical group with described aggressive behaviour, (**II**) in combination with a healthy control sample, (**III**) conducted during adolescence, (**IV**) reported whole brain gray matter volume alterations or whole brain functional neuroimaging data, (**V**) results are described using a standard reference space (Talairach or MNI) and (**VI**) the same threshold was used throughout the whole brain analysis. All structural studies included employed a standard VBM analysis protocol. In both meta-analysis of structural and functional brain alterations in adolescents with AB versus controls, no studies providing results based on a priori region-of-interest analysis only were included (since they violate the assumption, under the null hypothesis, that the likelihood of locating activated foci is equal at every voxel). Similarly, no animal studies or case reports were included in any meta-analysis and only studies from peer-reviewed journals that are written in English were considered. Data is current up to July 2015.

Of the 1021 studies identified through our systematic review (see Fig 1), we screened 930 (after removal of duplicates) and consequently assessed the full texts of 173 articles. 156 studies had to be excluded from the functional or structural meta-analysis in adolescents with AB, because they did not meet the criteria listed above (for detailed exclusion reasons, see Fig 1). Looking more closely at our review on *structural research studies* in AB revealed that only five studies reported on gray matter volume increases in AB (four reported de- and increases, one study only reported increases). Therefore we did not conduct a separate meta-analysis for gray matter volume increases in AB. Consequently, eight studies were included in our meta-analysis about gray matter volume reductions, together reporting data from 408 research participants (224 AB, 184 typically developing controls = TD), and 50 foci of gray matter volume decreases in youths with AB (Table 1 [21,22,23,66,67,68,69,70]).

**Table 1:** Characteristics of the studies in adolescents with AB included in the current structural meta-analysis.

*#*	*First author*	*Year*	*Method*	***Diagnosis [N]***	***Sex [m/f]***	***Average age and [range] in years***
1	**Huebner**	2008	VBM	CD, early-onset [23]	[23/0]	CD, early-onset: 14.5
	** **			TD [23]	[23/0]	TD: 14.2
	** **					[12–17]
2	**De Brito**	2009	VBM	CP/CU+ [23]		CP/CU+: 11.5
	** **			TD [25]		TD: 11.8
	** **					[10–13]
3	**Dalwani**	2011	VBM	CP+SUD [25]	[25/0]	CP+SUD: 16.6
	** **			TD [19]	[19/0]	TD: 16.6
	** **					[14–18]
4	**Fahim**	2011	VBM	DBD [22; 11CD/11ODD]	[22/0]	DBD: 8.4
	** **			TD [25]	[25/0]	TD: 8.4
	** **					
5	**Fairchild**	2011	VBM	CD, early-onset [36]	[36/0]	CD, early-onset: 17.7
	** **			CD, late-onset [27]	[27/0]	CD, late-onset: 17.9
	** **			TD [27]	[27/0]	TD: 18.5
	** **					[16–21]
6	**Stevens**	2012	VBM	CD [24]	[16/8]	CD: 16.0
	** **			TD [24]	[16/8]	TD: 16.0
	** **					[12–18]
7	**Fairchild**	2013	VBM	CD [22]	[0/22]	CD: 17.6
	** **			TD [20]	[0/20]	TD: 17.2
	** **					[14–20]
8	**Dalwani**	2015	VBM	CP [22]	[0/22]	CP: 16.7
				TD [21]	[0/21]	TD: 16.1
						[14–18]

CD = Conduct disorder. DBD = Disruptive behaviour disorders. CU+ = with high callous-unemotional traits. SUD = Substance use disorder. TD = Typically developing participants. VBM = Voxel-based morphometry.

Our systematic literature review of *functional neuroimaging studies* in youths with AB identified experiments targeting emotion processing [34,35,36,37,38,39,40,41,42,43], empathy [41,44,45], theory of mind [46], passive avoidance [47], decision making [48,49] or executive functioning [40,42,50]. We decided to restrict our functional meta-analysis to tasks only including emotionally loaded and visually presented stimuli (e.g. tasks of emotion processing and empathy). In case of sample overlap, the study with the highest subject number meeting all other criteria listed above was selected. In case of comparisons between AB and TD in more than one contrast, only foci from the contrast putting the highest demand on emotion processing, were included. The majority of studies indicated hypoactivations in AB. Only six studies that fulfilled all other criteria listed above reported hyperactivations in AB compared to TD. Therefore, we did not conduct a separate meta-analysis on functional overactivations in AB. Consequently nine studies suggesting hypoactivations in adolescents with AB compared to TD were selected (Table 2; [34,39,40,41,43,44,71,72,73]). Together the selected studies report data from 375 research participants (215 AB, 160 TD) and describe 58 foci of hypoactivation in AB compared to TD.

**Table 2:** Characteristics of the studies in adolescents with AB included in current functional meta-analysis.

#	***First author***	***Year***	***Stimuli***	***Diagnosis [N]***	***Sex [m/f]***	***Average age and [range] in years***
1	**Sterzer**	2005	Neutral or negative	CD [13]	[13/0]	CD: 12.9
	** **		**pictures** (IAPS)	TD [14]	[14/0]	TD: 12.7
						[9–15]
2	**Passamonti**	2010	**Pictures** of angry, sad	CD, early-onset [27]	[27/0]	CD, early-onset: 17.7
			and neutral faces	CD, late-onset [25]	[25/0]	CD, late-onset: 17.1
				TD [23]	[23/0]	TD: 17.8
						[16–21]
3	**Marsh**	2011	Emotional **words**	CD/ODD+PT [14]	[8/6]	CD/ODD+PT: 14.4
			(categorization task)	TD [14]	[11/3]	TD: 13.5
						
4	**White**	2012	**Pictures** of fearful and	CD/ODD+PT [15]	[12/3]	CD/ODD+PT: 15.7
	** **		neutral faces	TD [17]	[9/8]	TD: 14.5
						[10–17]
5	**Lockwood**	2013	**Pictures** of others in	CD [37]	[37/0]	CD: 14.05
			pain or no pain	TD [18]	[18/0]	TD: 13.68
						[10–16]
6	**Marsh**	2013	**Pictures** of others in	CD/ODD+PT [14]	[8/6]	CD/ODD+PT: 15.4
			pain or no pain.	TD [21]	[15/6]	TD: 14.3
						[10–17]
7	**Fairchild**	2014	**Pictures** of emotional	CD [20]	[0/20]	CD: 17.0
			or neutral faces	TD [20]	[0/20]	TD: 17.6
						
8	**O'Nions**	2014	Cartoons (affective	CP/CU+ [16]	[16/0]	CP/CU+: 14.2
			**picture** series)	TD [16]	[16/0]	TD: 13.5
						[10–16]
9	**Sebastian**	2014	**Pictures** of fearful and	CP/CU+ [17]	[17/0]	CP/CU+: 14.0
			calm facial expressions	CP/CU- [17]	[17/0]	CP/CU-: 14.5
				TD [17]	[17/0]	TD: 13.5
						[10–16]

CD = Conduct disorder. CP = Conduct problems. ODD = Oppositional defiant disorder. PT = with psychopathic traits. CU+ = with high callous-unemotional traits. CU- = with low callous-unemotional traits. TD = Typically developing participants.

### ALE Meta-Analysis Procedure

We conducted two separate meta-analyses on gray matter volume alterations and functional hypoactivations in adolescents with AB. Data analysis was carried out using the revised version of the ALE approach for coordinate-based meta-analysis of neuroimaging data (GingerALE software, version 2.3; available from http://brainmap.org/ale/ [51,74,75,76]). In short, this new approach implements a random-effects model, a quantitative uncertainty model to determine the FWHM and an exclusive gray matter mask (for further details, see also [51,53,74,75,76]). Most importantly, instead of testing for an above-chance clustering between foci, the revised ALE algorithm assesses above-chance clustering between experiments. The spatial relationship between foci in a given experiment is now assumed to be fixed and ALE results are assessed against a nulldistribution of random spatial association between experiments. Prior to running any analyses, coordinates reported in Talairach space were transformed to MNI space using the tal2icbm algorithm [77,78]. The here employed revised ALE approach identifies areas of convergence of activation across various experiments, minimizing the within-groups effects (approach by Turkeltaub and colleagues [75]). Each focus is represented as a centre for 3D Gaussian probability distributions, where the standard deviation depends on group size (capturing spatial uncertainty) rather than single time points. First, the probabilities of all activation foci in a given experiment are combined for each voxel, which is represented in modelled activation maps (fMRI) or modelled anatomical maps (VBM). Secondly, the ALE method combines all modelled maps (fMRI and VBM separately) on a voxel-by-voxel basis to form an ALE image containing all unthresholded voxel ALE values. In the last step, this ALE image is tested against the null hypothesis under the assumption that all activated voxels are homogeneously distributed in the brain, independent of the experiments. This null-hypothesis model (a distribution map made by multiple permutations of random voxel activation) was created using a random-effects statistical method and tested against the original ALE image according to the selected significance threshold. Therefore, the nulldistribution is constructed reflecting a random spatial association between different studies. Comparing the “true” ALE score to this distribution allows a focused inference on convergence between studies while preserving the relationship between individual foci within each study. Critically, this change from fixed- (foci-based) to random-effects (testing between study effects) inference in ALE analysis allows generalisation of the results to the entire population of studies from which the analysed ones were drawn. This more conservative approach with an increased specificity [51,76] does also accommodate the idea of convergence across heterogeneous studies. We used a statistical threshold of p<0.05 False Discovery Rate (FDR) corrected for multiple comparisons and a minimum cluster size of 500mm^3^. ALE maps are overlaid onto a standard brain in MNI space (Colin27 available at http://www.brainmap.org/ale/) using the Multi-image Analysis GUI (Mango available at http://ric.uthscsa.edu/mango/mango.html) and clusters were anatomically labelled by cross-referencing the Talairach Daemon [79,80] and aal [81]. In order to further investigate possible overlaps between the structural (VBM) and functional (fMRI) meta-analysis in adolescent AB, a formal conjunction analysis was performed by multiplying binarized versions of the individually thresholded ALE maps.

## Results

**Fig 2:**
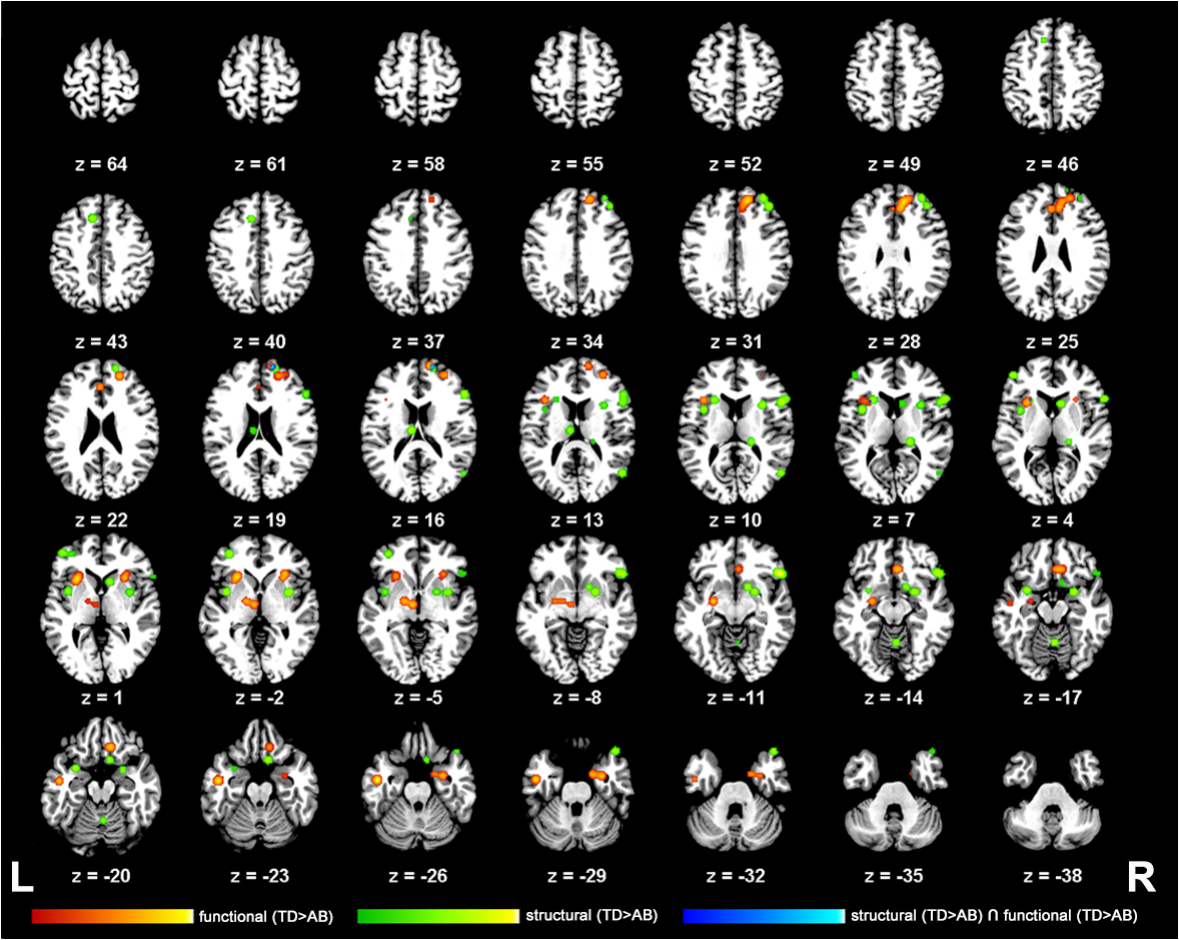
Neuronal alterations in adolescents with aggressive behaviour (TD>AB): Results from an ALE meta-analysis. 2-D axial slices displaying the thresholded and binarized ALE maps of significant overlap (P<0.05, FDR-corrected) in studies of structural (green) and functional (red) alterations in adolescent AB (TD>AB) as well as a conjunction analysis (blue) overlaid on the Colin T1-template in MNI space. Z-slices depicting the results range from z = 21 to 120 and are displayed in neurological view using the Multi-image Analysis GUI (Mango available at http://ric.uthscsa.edu/mango/mango.html).

Our meta-analysis of *structural neuroimaging studies* in adolescents with AB revealed 19 clusters of significant convergence between the studies (see Table 3; Fig 2). The largest clusters were found in the right inferior frontal lobe (inferior frontal/precentral gyrus), right precuneus and left-hemispheric insula. Further smaller clusters were found bilaterally in the frontal (e.g. dorsolateral and medial frontal gyrus), parietal (e.g. precuneus) and temporal lobe (e.g. middle/superior temporal gyrus) as well as the cerebellum (e.g. culmen). Our meta-analysis of *functional hypoactivation* in adolescents with AB revealed 8 clusters of significant convergence between the studies with the largest clusters in the right middle/superior frontal gyrus, left thalamus and basal ganglia, as well as left-hemispheric insula (see Table 3, Fig 2). Beyond others, further clusters included the right anterior cingulate, left middle temporal gyrus and right amygdala.

**Table 3:** Results of the structural and functional ALE-meta analyses and conjunction analysis of structure and functional alterations in adolescents with AB.

** **	**Region**	**BA**	**H**	**Volume**		**Local Maxima**	** **
*Structural Meta-Analysis (TD>AB)*							
1	inferior frontal/precentral gyrus,	13,	R	1952	54	16	10
	insula	44, 45			62	20	6
					56	26	16
2	subcallosal gyrus, putamen,	34	R	1672	26	4	-16
	lateral globus pallidus, amygdala				22	4	-8
					14	10	-12
3	inferior frontal gyrus	45, 47	R	1304	52	26	-10
4	insula	13	L	1144	-38	8	8
					-38	4	-2
5	middle/superior frontal gyrus	9,8	R	1112	34	48	30
					40	38	30
6	middle/inferior frontal gyrus	10,46	L	1040	-36	48	-2
					-46	48	2
7	putamen, claustrum		R	688	34	2	-2
8	thalamus		R	560	20	-30	8
9	subcallosal/middle frontal gyrus, cingulate	25	R	528	10	14	-22
10	cingulate/middle frontal gyrus	32	L	528	-10	24	42
11	claustrum		L	520	-24	20	8
12	claustrum, insula		R	520	32	14	10
13	subcallosal/parahippocampal gyrus, amygdala	34	L	512	-30	4	-18
14	culmen, declive		R	512	4	-58	-16
15	caudate		R	512	10	14	2
16	thalamus		L	512	-8	-16	15
17	inferior frontal gyrus	47	R	504	46	26	-30
18	middle temporal gyrus	37	R	504	54	-68	12
19	superior frontal gyrus	9	R	504	18	56	20
	All x, y, z-coordinates represent local maxima in MNI space				AB=Aggressive Behaviour		
	Volume=Volume (mm^3^)				TD=Typically developing controls		
	H=Hemisphere				BA= Brodmann areas		
	R=Right; L=Left						
** **	**Region**	**BA**	**H**	**Volume**		**Local Maxima **	
*Functional Meta-Analysis (TD>AB)*							
1	middle/superior frontal gyrus,	8, 9,	R/L	3728	14	44	30
	anterior cingulate gyrus	10, 32			8	36	28
					22	48	22
					32	50	14
					0	36	24
2	thalamus, lentiform nucleus,		L	1944	-6	-12	-4
	putamen, medial globus pallidus				-26	-8	-12
	amygdala				-16	-8	-4
3	claustrum, insula	13	L	1896	-28	20	0
					-38	20	12
4	middle frontal gyrus,	11, 24	R	1328	12	30	-20
	anterior cingulate				4	30	-14
5	inferior/middle temporal gyrus	21	L	1288	-48	-8	-26
6	amygdala, parahippocampal	28	R	1224	30	-4	-28
	gyrus				20	-2	-30
7	claustrum, putamen, insula	13	R	776	28	20	0
					30	24	-2
8	superior, middle frontal gyrus	9	R	552	14	60	16
*Conjunction*: *Structural (TD>AB)* ∩ Functional (TD>AB)							
1	superior frontal gyrus (dmPFC)	9	R	128	16	58	18
2	claustrum, insula		L	8	-26	20	4
3	claustrum, insula		L	8	-28	18	6

All x, y, z-coordinates represent local maxima in MNI space. AB = Aggressive Behaviour. Volume = Volume (mm3). TD = Typically developing controls. H = Hemisphere. BA = Brodmann areas. R = Right; L = Left.

**Fig 3:**
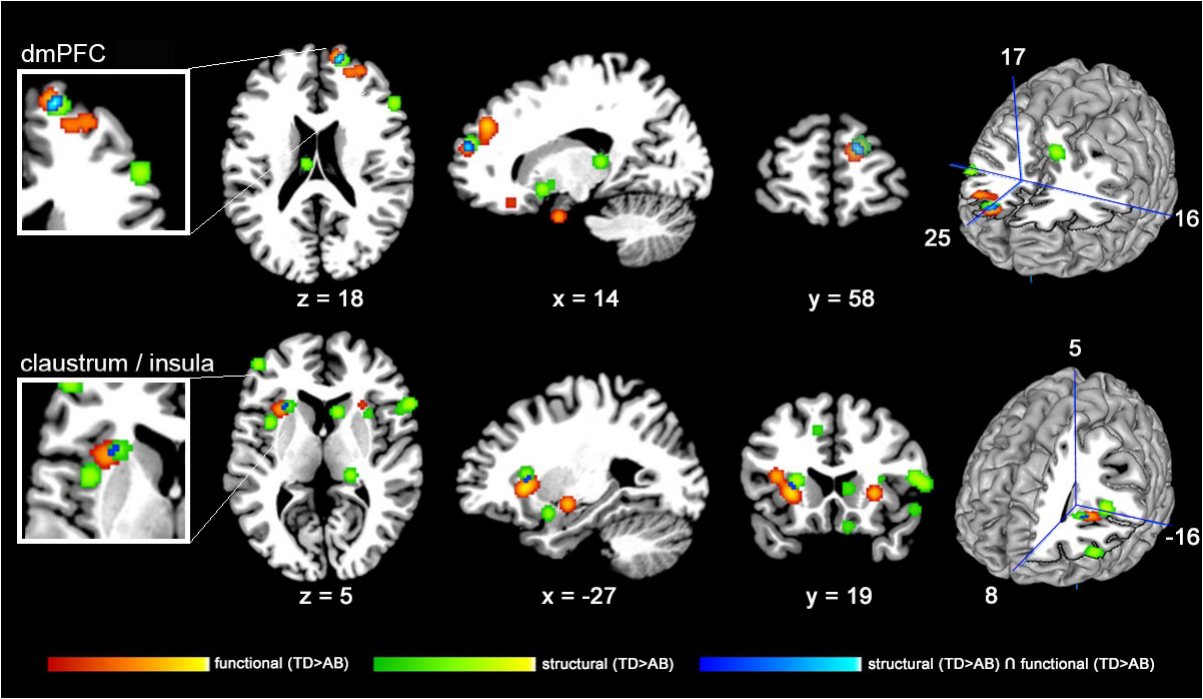
Structural and functional neuroimaging findings in youths with AB co-localize in right dorsomedial prefrontal cortex (dmPFC) and left insular cortex. 2-D slices displaying the thresholded and binarized ALE maps of significant overlap (P<0.05, FDR-corrected) in studies of structural (green) and functional (red) alterations in adolescents with AB (TD>AB) as well as a conjunction analysis (blue) overlaid on the Colin T1-template in MNI space. The upper-row including left cut-out as well as right surface-model highlight the right dmPFC where structural and functional alterations co-localize. The lower-row including left cut-out as well as right surface-model illustrate left insular cortex/claustrum where structural and functional alterations overlap.

A formal conjunction analysis using the thresholded ALE maps from the structural and functional meta-analysis discovered three areas of regional overlap (Table 3, Fig 3 **)**. The biggest area of functional and structural overlap (128mm^3^) in adolescents with AB was identified within the right dmPFC. Additionally, the analysis exposed two smaller, close-lying clusters of convergence with a peak in the left claustrum, extending into the insular cortex.

## Discussion

To our knowledge, the current work provides the first quantitative summary of functional hypoactivations and gray matter volume reductions in adolescents with AB by summarizing findings of eight structural and nine functional neuroimaging studies in a total of 783 participants (408 [224 AB/184 TD] and 375 [215 AB/160 TD] for structural and functional analysis respectively). Our findings indicate 19 structural and eight functional foci of significant alterations in AB, mainly located within the emotion processing and regulation network of the human brain (including orbitofrontal, dorsolateral/medial prefrontal cortex and limbic brain regions; for reviews on emotion processing and regulation see also [82,83,84]). Conjunction analysis reveal that functional and structural alterations in AB overlap in three areas, with the largest cluster centered in the right dmPFC and two smaller clusters that encompass the left insula.

In the following sections we will review structural and functional neuroanatomical evidence derived from healthy participants as well as those with aggressive behaviour (e.g. conduct problems, CD, ODD) for the key areas implicated here (orbitofrontal and dorsomedial prefrontal cortex, insula, cingulate cortex, amygdala).

### Orbitofrontal and Dorsomedial Prefrontal Cortex

Our findings identify prefrontal brain regions including orbitofrontal and dorsomedial prefrontal cortex as main locations of aberrant brain function and structure in youths with AB. Furthermore, an overlap in the foci representing structural and functional changes that co-localize in AB is centered in the right dmPFC. While the orbitofrontal as well as the dorsomedial prefrontal cortex can be differentiated based on quantitative as well as qualitative markers [85], both have equally been suggested in emotion processing and working memory/inhibitory control [86]. The medial prefrontal cortex in particular has been implicated in emotional self-regulation [87], general self-referential activities [88] and emotion-related decision making [89]. Meta-analytic evidence suggests a more generic role of the dmPFC in emotion processing (e.g. appraisal, evaluation, experience, response), non-specific to a particular emotion [90]. In addition, lesion, neurophysiological and neuroimaging evidence have linked the orbitofrontal and dorsomedial prefrontal cortex to stimulus-reinforcement association learning [91]. The ability to rapidly decode and readjust values of different input signals is likely to be crucial to emotional behaviour and may ultimately influence emotional learning. It has been suggested that the observed deficits in decision making may directly result from aberrant emotion processing as for example observed after frontal brain damage [91]. Research has for instance demonstrated that aberrant self-monitoring abilities may be responsible to preclude the generation of social emotions typically associated with the resolution of social mistakes [92]. Finally, a whole line of evidence (e.g. [18,93,94]) has linked the prefrontal cortex to aggression. In its extreme, antisocial personality disorder and psychopathy are exemplary for individuals displaying increased aggressive behaviour and studies of both have linked structural [95,96] and functional [97,98] changes to the prefrontal cortex.

### Insula

Both our functional and structural AB meta-analysis have found significant clusters of hypoactivations or altered brain structure within the insula. In addition to that, two smaller clusters reached significance in the left insular cortex during our conjunction analysis, mapping structural and functional alterations in youths with AB. The insula or insular cortex is part of the cerebral cortex forming the base of the lateral sulcus (or sylvian fissure [99]). From a neurodevelopmental perspective it is the first region of the cortex to develop and differentiate around 6 weeks of fetal life [100]. The insula is bi-directionally connected to various brain regions, including the orbitofrontal cortex, anterior cingulate, supplementary motor areas, parietal and temporal cortices, but also to subcortical structures such as the amygdala, basal ganglia and thalamus [99,101]. Connectivity to and from the insula is divided, in that the anterior part of the insula has greater connectivity with the frontal lobe, while posterior parts are more strongly connected to the parietal lobe. Neuroimaging evidence has suggested that the insula may play a key role in the awareness of bodily sensations and affective feelings [84,102]. Meta-analytic data supports this idea, and suggests that the insula is a key player in the evaluation, experience or expression of internally generated emotions [90]. Particularly the left insula, along with frontal and temporal brain regions, is associated with anger [84]. Furthermore, an emotion-specific role of the insula for disgust [103] has been discussed. However, the majority of neuroimaging findings and meta-analytic reviews to date support a generic role of the insula in emotional behaviour (e.g. [84,104]).

Atypical neuronal functioning of the insula (e.g. during tasks of emotion processing and empathy) are linked to AB (e.g. [41,97]). However, so far, both hyper- [45,71] and hypoactivations [39,41,105] are observed during tasks of empathy, face or pain processing. In psychopathy particularly fear conditioning has been linked to aberrant insula activation [106]. Functional atypicalities within the insula are further observed in borderline personality disorder [107], schizophrenia [108], depression [109] or anorexia nervosa [110]. Gray matter volume alterations within the insula are associated with various psychiatric conditions beyond antisocial populations (e.g. [24,96]), including bipolar disorder [111], schizophrenia [56], drug dependence [112], major depression [113] or anorexia nervosa [114].Therefore, the neuronal and structural alterations within the insula may reflect a characteristic of psychiatric conditions per se [99].

### Cingulate Cortex

The cingulate cortex showed functional as well as structural foci of significance in each of our two meta-analyses individually. Cytoarchitectonically, the cingulate gyrus may be divided into four functionally independent but interconnected subregions, including the anterior cingulate cortex (emotion), the midcingulate cortex (response selection), the posterior cingulate cortex (personal orientation), and the retrosplenial cortex (memory formation and access) [115]. Overall the cingulate cortex has been implicated in the regulation of cognitive as well as emotional processes [90,115] (e.g. processing of acute pain [116] or affective stimulus material [115]), most likely through an interaction with the prefrontal cortex, anterior insula, premotor area, the striatum and cerebellum [115,117]. We here particularly identified regions within the bilateral anterior cingulate as foci of interest through both our functional and structural meta-analysis. While dorsal aspects of the anterior cingulate have been linked to tasks of executive functioning [118,119], the anterior part of the cingulate is part of the emotion processing network [119,120]. It is further suggested that the cingulate gyrus may serve as a transition and/or interaction zone between affective and cognitive processing [90].

Studies in AB and antisocial personality disorder have found both gray and white matter increases as well as decreases within the cingulate (e.g. [23,68,121,122]); the developmental pathway within this region thus still needs further assessment. Hypoactivation in AB within the cingulate has been reported during tasks of emotion processing [34,35], empathy [41,67], response inhibition [85] and sustained attention [105]. Similarly, individuals with antisocial personality disorder or psychopathic tendencies show reduced activation within the cingulate during tasks of emotion processing and conflict resolution, as for example observed in moral decision making [123,124], deception [125], frustration [126] and emotion processing [127].

### Amygdala

Both our functional and structural meta-analyses have identified the right and left-hemispheric amygdala as significant foci of interest, even though this area has not reached significance in our conjunction analysis. The amygdala is crucial for the perception and encoding of emotionally loaded stimulus material and has been suggested as the brain locus of fear (e.g. detection, generation, maintenance of fear and coordination of response in the danger of such) [84,128]. To summarize the existing fMRI evidence, neuronal activation within the amygdala has been observed in healthy individuals in tasks that include arousing stimulus material (e.g. emotionally loaded images [129,130], facial expressions [131,132,133] or words [134,135]), during tasks of empathy [136,137], moral reasoning [138] or when processing potential threats [139]). A range of tasks investigating amygdala responses to different evocative stimulus material led to the suggestion that increased activation within the amygdala may particularly mirror affective processing under acute danger or threat, rather than fear per se [90]. Furthermore, neuronal activation is thought to mirror dispositional affective style [90,140], whereby increased amygdala activity correlates with affective reactivity to negative stimuli. Interestingly, amygdala activation in response to emotionally loaded stimuli may be attenuated by task demand [120,141,142] or comorbid anxiety and depression symptoms [34]. For example, concurrent goal-directed processing can disrupt amygdala activation that is evoked by emotional images [142]. This is in line with meta-analytic evidence indicating that studies employing a cognitive task during affect processing are less likely to demonstrate amygdala activation [90].

Because of its role in aversive conditioning, instrumental learning and fear processing, the amygdala is often chosen as a region of interest in investigations targeting AB, antisocial personality disorder or psychopathy [18]. Amygdala dysfunction is suggested to be one of the core features in the symptomatology of antisocial disorders (e.g. [18,34,43,143]). Structurally, the amygdala is altered in AB similarly as in antisocial personality disorders and psychopathy (e.g. [24,144,145]). Finally, it is to note that the amygdala is strongly interconnected with the orbitofrontal brain regions and alterations in the connectivity between these two centers have been reported in AB and psychopathy (e.g. connectivity between key regions of the emotion processing and regulation network (e.g. [146,147], for a further discussion see following section).

### Structure-Function Relationship and Connectivity Findings

While neuroplasticity is known to potentially range from synaptic plasticity to more complex changes (e.g. shrinkage in cell size, neural or glial cell genesis, spine density or even changes in blood flow or interstitial fluid [148]), the neurophysiological basis of experience-induced neuroplasticity is still a matter of extensive research [14]. Some studies indicate that functional and structural measures of plasticity may be related. For example it could be hypothesized that experience-related gray matter volume changes correspond to task-specific processing, or, more precisely, synaptic remodelling within specific processing areas [149]. Another possibility may be that impaired connectivity between key regions leads to the functional alterations observed. For example researchers have argued that the social and emotional deficits seen in AB may be mediated by impaired connectivity between the emotion processing and regulation network [146,147]. These system-specific deficits may be observed by diffusion tensor imaging and tractography measurements. For example, the uncinate fasciculus is a white-matter tract connecting the amygdala and neighbouring anterior temporal lobe with the orbitofrontal cortex and it thus may be involved in facilitating empathy, emotion regulation and socio-cognitive processes [150]. Such models would for example explain why local changes in brain structure cannot always be inferred from purely functional models. For example in individuals with reactive aggression aberrant amygdala activity but intact amygdala structure is observed [151]. In such cases it is possible that impaired fibre connections (e.g. reduced functional anisotropy in the uncinate fasciculus) to and from this area cause the neuronal differences observed [151]. In line with evidence in AB [151] significant differences in the fractional anisotropy (FA) measures of the uncinate fasciculus have been demonstrated in adolescents with conduct disorder [29,39] as well as in adult psychopathy [144,152]. Similarly, studies of intrinsic connectivity (resting state) explore functional networks that are non-stimulus driven and may inform about the basic functional brain architecture while implicating anatomical connectivity of the regions involved [153]. In individuals with antisocial personality disorder this intrinsic connectivity between highly interconnected brain centres is disrupted [154].

Independent of the precise neurophysiological nature of structure-function associations, our results have indicated co-localized structural and functional deficits in right dmPFC and left insular cortex. Based on today’s structure-function knowledge we thus hypothesize that decreased synaptic density may have led to a co-localized decrease within the BOLD response measured through fMRI. However, it has to be noted that here we only investigate co-localized structure-function findings that are based on gray matter volume reductions and functional hypoactivations in AB. This limitation (no volume increases or hyperactivity investigated) is due to the nature of the existing neuroimaging evidence, with only five studies reporting gray matter volume increases and six studies providing evidence for functional hyperactivations in individuals with AB. Further studies comparing adolescents with AB compared to controls are needed in order to examine functional hypoactivations and gray matter volume increases more extensively. Furthermore, only longitudinal research studies will be able to show the precise developmental trajectory of these alterations in detail.

### Limitations

Meta-analytic approaches such as the current one have a number of limitations in need for discussion. The presented analyses are first of all limited by the detail and quality of the original research studies. This includes problems of variations within the significance threshold of data reported, insufficient information on possible coordinate transformations and variation in group sizes. Additionally, even though psychosocial factors have been significantly linked to brain structure in AB, none of the studies to date systematically studied the influence of these within their designs. Furthermore, only a small number of studies to date have examined brain structure and function in youths with AB on a whole brain level. We decided that a more stringent inclusion criteria is beneficial over the absolute number of studies entering the analyses, especially in regards to the attempt to truly capture the neuronal and structural phenotype of adolescents with AB. The number of studies entering each analysis therefore is on the lower limit. Contrast analyses are ideally contain a minimum of 15 studies in each dataset to obtain sufficient statistical power (http://brainmap.org/ale/ [51,74,75,76]). Therefore, the current analysis runs the risk of being under-powered.

Most of the studies included here consisted of only, or majority of, male participants (see Tables 1 and 2). Some of the included study designs considered sex-matched clinical and control groups, while others applied a gender covariate within their design (e.g. [26,69]). Two VBM [21,70] and one fMRI [71] study included only female participants. These studies were nevertheless included in the current meta-analyses because the structural alterations observed in girls with CD broadly overlapped with those previously reported in male samples only [21]. But while the current population included mirrors the occurrence of AB in the general population (e.g. higher number of males with AB [19]), research has shown that it may be crucial to differentiate clinical cases based on gender in future research studies (e.g. [155]). Specifically, to determine possible gender related differences of structural and functional characteristics in individuals with AB, a comparison between meta-analyses of studies examining females and those examining males separately would have been of interest, but was not possible due to the small number of studies that are available for each group individually.

Another potential caveat is the fact that clinical and subclinical forms of aggressive behaviour are often associated with comorbid diagnoses, most prominently attention-deficit hyperactivity disorder (ADHD; reported in up to 69% of CD patients [156]) and anxiety [19]. To date there is no neuroimaging evidence investigating pure diagnosis of clinical manifestations of aggressive behaviour (e.g. CD or ODD) [157]. Researchers argue whether aggressive behaviour in combination with ADHD even posits a distinct subtype or not [158] and common neurobiological pathways are considered [157]. Overall it can be concluded that neuroimaging research studies on aggressive behaviour in children and adolescents to date are characterized by diverse approaches in regards to the sample selection and definition, all of which have their justification and pitfalls [159]. Ultimately, only a comparisons of both, pure and comorbid groups will be able to inform about the specificity and predictive value of either definition. Here we included adolescents with clinical and subclinical forms of aggressive behaviour, most of which have comorbid ADHD symptoms (e.g. [21,22,23,26,34,39,40,41,43,44,66,67,71,72,73]. Many of the included studies report no differences in results when controlling for ADHD (through exclusion or a covariate within the study design; [34,39,43,44,48,71,72]).

Similar problems are IQ differences, drug use or socioeconomic status, all of which are a characteristic of populations with aggressive behaviour. Studies included in the current meta-analysis have all matched their participants according to IQ measures [22,39,40,41,43,44,66,68,69,71,72,73] or used IQ as a covariate within their study design [21,23,25,26,34,67]. Drug use and socio-economic status were controlled for in some, but not all, studies and further research is needed using a more careful sample characterisation in order to inform about the impact of these variables on brain structure and function.

It is also to consider that the diagnosis of conduct disorder (clinical manifestation of AB) may encompass at least two clinically relevant subgroups. While the first group exhibits callous-unemotional traits (e.g. reduced guilt, callousness, uncaring behaviour and reduced empathy) and heightened risk of persistent antisocial behaviour, the second group is characterized by heightened threat sensitivity and reactive aggression [1,160]. Callous-unemotional traits are highly heritable [161], expressed as early as at two years of age [162] and are predictive of the most severe and persistent variant of conduct disorder [163,164]. Studies also indicate that this severity may significantly impact the neuronal alterations observed [22,39,71,165]. To summarize, while we were unable to constrain the current meta-analysis based on potential subtypification and gender variables, these factors may pose an exciting view on data analysis strategies and interpretations for future studies. For all the reasons noted, the current results have to be interpreted with caution. However, multimodal neuroimaging methods combining two or more functional (fMRI and/or EEG) and structural (MRI and/or DTI) approaches are suggested to provide a more sensitive measure in comparison to unimodal imaging for disease classification [166]. Furthermore, we think that the confounding variables discussed here have influenced the functional and structural meta-analyses similarly.

Overall, we could demonstrate that structural and functional alterations in adolescents with AB co-localize within key regions of the emotion processing and regulation network (e.g. prefrontal and insular cortex). Thus, our current analysis, using an activation likelihood estimation approach, provides an important step towards a more focused method of neuroimaging in AB. Future studies need to determine whether the here identified convergent clusters of neuronal and structural alterations may be applicable for clinical purposes (for example an improved pathophysiological description of individuals with AB) or whether a further specification (e.g. based on subtypes and gender) may be needed. However, the coordinates presented here can serve as non-independent regions of interest for future studies in AB, conduct disorder or in individuals with AB or antisocial/psychopathic tendencies.

## Summary and Conclusion

Aggressive behaviour constitutes a major issue of public health and increased knowledge about the behavioural and neuronal underpinnings of AB are crucial for the development of novel and implementation of existing treatment strategies. However, single site studies often suffer problems of small sample size and thus power issues. Quantitative meta-analysis techniques using activation likelihood estimations as implemented here offer a unique opportunity to investigate consistency of results between several studies investigating the same research question and population. We have implicated several brain regions of the emotion processing and regulation network to show hypoactivations and gray matter volume reductions in adolescents with AB (including prefrontal brain regions, amygdala, insular and cingulate cortex) and demonstrated that functional and structural alterations in AB co-localize within right dmPFC and left insular cortex.

Overall, we are in line with meta-analytic work as well as structural, functional and connectivity findings that make a strong point for the involvement of a network of brain areas responsible for emotion processing and regulations. This network is impacted in individuals with AB and antisocial personality disorder/psychopathy. However, much still needs to be investigated. For example, study findings differ in regards to hypo- or hyperactivations and gray matter volume reductions or increases in different regions of the emotion processing and regulation network. Due to power constraints, the current meta-analysis only investigated hypoactivations and gray matter volume reductions in youths with AB and no hyperactivations or increases in brain structure. Future studies implementing longitudinal designs may be able to shed more light on the developmental pathway as well as onto typical and atypical trajectories within the regions reported. Such longitudinal designs will further allow the investigation of the bidirectional influence of biological and psychosocial influences in AB.

## Supporting Information

S1 Table Checklist for PRISMA items. (DOC)
